# Calibrating animal‐borne proximity loggers

**DOI:** 10.1111/2041-210X.12370

**Published:** 2015-05-06

**Authors:** Christian Rutz, Michael B. Morrissey, Zackory T. Burns, John Burt, Brian Otis, James J. H. St Clair, Richard James

**Affiliations:** ^1^ Department of ZoologyUniversity of OxfordSouth Parks RoadOxfordOX1 3PSUK; ^2^ School of BiologyCentre for Biological DiversityUniversity of St AndrewsSir Harold Mitchell BuildingSt AndrewsKY16 9THUK; ^3^ Department of Electrical EngineeringUniversity of WashingtonSeattleWA98195USA; ^4^ Department of Physics and Centre for Networks and Collective BehaviourUniversity of BathBathBA2 7AYUK; ^5^ Present address: School of BiologyCentre for Biological DiversityUniversity of St AndrewsSir Harold Mitchell BuildingSt AndrewsKY16 9THUK

**Keywords:** animal social network, biologging, business card tag, contact network, *Corvus moneduloides*, direct and indirect encounter mapping, Encounternet, reality mining, transceiver tag, wireless sensor network

## Abstract

1. Growing interest in the structure and dynamics of animal social networks has stimulated efforts to develop automated tracking technologies that can reliably record encounters in free‐ranging subjects. A particularly promising approach is the use of animal‐attached ‘proximity loggers’, which collect data on the incidence, duration and proximity of spatial associations through inter‐logger radio communication. While proximity logging is based on a straightforward physical principle – the attenuation of propagating radio waves with distance – calibrating systems for field deployment is challenging, since most study species roam across complex, heterogeneous environments.
2. In this study, we calibrated a recently developed digital proximity‐logging system (‘Encounternet’) for deployment on a wild population of New Caledonian crows *Corvus moneduloides*. Our principal objective was to establish a quantitative model that enables robust *post hoc* estimation of logger‐to‐logger (and, hence, crow‐to‐crow) distances from logger‐recorded signal‐strength values. To achieve an accurate description of the radio communication between crow‐borne loggers, we conducted a calibration exercise that combines theoretical analyses, field experiments, statistical modelling, behavioural observations, and computer simulations.
3. We show that, using signal‐strength information only, it is possible to assign crow encounters reliably to predefined distance classes, enabling powerful analyses of social dynamics. For example, raw data sets from field‐deployed loggers can be filtered at the analysis stage to include predominantly encounters where crows would have come to within a few metres of each other, and could therefore have socially learned new behaviours through direct observation. One of the main challenges for improving data classification further is the fact that crows – like most other study species – associate across a wide variety of habitats and behavioural contexts, with different signal‐attenuation properties.
4. Our study demonstrates that well‐calibrated proximity‐logging systems can be used to chart social associations of free‐ranging animals over a range of biologically meaningful distances. At the same time, however, it highlights that considerable efforts are required to conduct study‐specific system calibrations that adequately account for the biological and technological complexities of field deployments. Although we report results from a particular case study, the basic rationale of our multi‐step calibration exercise applies to many other tracking systems and study species.

## Introduction

The structure of animal social networks has profound consequences for a wide range of phenomena, including the transmission of genes, pathogens and social information (reviews: Croft, James & Krause 2008; Whitehead 2008; Kurvers *et al*. 2014; Pinter‐Wollman *et al*. 2014). In the majority of cases, researchers infer social networks from data on the spatial grouping of study subjects. Two individuals are considered to ‘associate’ with or ‘encounter’ one another (and would therefore form an ‘edge’ in a network), if they were seen together within a predefined distance, over which the biological process of interest can operate (for an example, see below). In some study systems, robust results can be obtained through repeated resightings of naturally or artificially marked subjects. But, for many other species, collecting even this most basic type of data in the wild is impossible, because they avoid human observers or range across inaccessible habitats. Furthermore, even when resightings are feasible, observation frequencies are usually insufficient (once per day, week or month), to enable analyses of fine‐scale patterns. Much higher sampling rates are required (in the order of once per hour or minute), to fully explore the biological causes and consequences of dynamically changing network topologies (Blonder *et al*. 2012; Krause *et al*. 2013; Pinter‐Wollman *et al*. 2014; Rands 2014; Sih & Wey 2014).

To overcome these methodological constraints, researchers working on a wide range of study systems have started exploring the use of automated tracking systems that collect association data with the help of animal‐attached devices (for a comprehensive review, see Table 1 in Krause *et al*. 2013). A particularly promising approach is ‘proximity logging’, which employs wireless sensor network (WSN) technology for data collection, and in some cases, remote data transfer (mammals: e.g. Ji, White & Clout 2005; Douglas, Ji & Clout 2006; Prange *et al*. 2006; Böhm *et al*. 2008; Hamede *et al*. 2009; Meise *et al*. 2013; Weber *et al*. 2013; birds: Rutz *et al*. 2012). Animal‐mounted proximity loggers (henceforth also called ‘tags’, for simplicity) are miniature ‘transceivers’ that, unlike conventional radio beacons, act both as transmitters *and* receivers of radio signals; thus, whenever two animals come to within detection range, their tags exchange radio signals and ‘log’ encounter information in their on‐board memory. As explored in detail in this paper, proximity logging exploits the fact that radio waves attenuate predictably with distance; all other things being equal, two animal‐mounted tags in close proximity will exchange stronger radio signals than two tags that are farther apart (but see below). Signal‐strength values therefore contain information about tag‐to‐tag distance, from which encounters between tagged animals can be inferred. In some cases, proximity loggers only record binary (yes/no) encounter data (e.g. tags from Sirtrack Ltd., Hastings, New Zealand), where detection settings can be tuned either by reducing the tags’ transmission power or by programming them to ‘ignore’ signals below a certain threshold signal strength. In others, they record all received data, enabling *post hoc* filtering by signal strength at the analysis stage (‘Encounternet’ tags from Encounternet LLC, Washington, Seattle, USA). Whatever the particular settings, all systems require careful calibration before field deployment: only after a robust relationship has been established between signal strength and tag‐to‐tag (and hence animal‐to‐animal) distance can proximity‐logging data be used to identify biologically relevant encounters, and ultimately, to construct informative association networks.

**Table 1:** Hypothetical encounter ‘logs’ of Encounternet proximity loggers. ‘this.ID’ and ‘enc.ID’ are the identities of the receiving and transmitting tags, respectively; ‘first.time’ and ‘last.time’ are times (in 1/64 second ‘ticks’) of the first and last pulse received in a sequence; the following three ‘RSSI’ columns give signal‐strength statistics for the pulse sequence making up the encounter; and ‘type’ codes distinguish, among other things, tag‐to‐tag logs from error messages and masternode commands. Note that the first three rows show logs from tags programmed to record individual radio pulses, so that values for minimum, maximum and mean RSSI are identical (as in our calibration field experiments of Step 1); the final three rows, on the other hand, show logs from tags which were programmed to average across multiple consecutively received pulses (see main text), so that all values differ

this.ID	enc.ID	first.time	last.time	RSSI.max	RSSI.min	RSSI.mean	type
61	42	1657379393	1657379393	7	7	7	1
22	42	1656954354	1656954354	−19	−19	−19	1
56	59	1654468502	1654468502	11	11	11	1
78	56	1657907367	1657927837	−14	−19	−15	1
10	61	1657315923	1657317204	8	1	4	1
38	54	1654582110	1654601313	17	−20	−4	1

The relationship between distance and signal strength is noisy in the real world, where internal, tag‐related factors (such as variation in transmission power due to current spikes), and external, environmental ones (such as humidity; see Marfievici *et al*. 2013), can cause considerable variation in radio transmission even between static tags; any attempt to infer inter‐tag distance from signal strength must necessarily be probabilistic. Several studies have reported calibration data for proximity‐logging tags with binary data recording and relatively short detection ranges of up to a few metres (e.g. Drewe *et al*. 2012; Boyland *et al*. 2013). Experiments have explored, among other things, the effects of habitat (Böhm, Hutchings & White 2009), logger attachment (Hamede *et al*. 2009), antenna alignment (Prange *et al*. 2006) and even subtle differences in tag performance (Boyland *et al*. 2013). Here we describe a comprehensive calibration for a novel proximity‐logging technology (‘Encounternet’) that, uniquely among commercially available systems, is capable of recording raw signal‐strength data for animal encounters over a wide range of distances, up to several tens of metres (Rutz *et al*. 2012; Meise *et al*. 2013) – features that will greatly enhance researchers’ ability to investigate biological processes that can operate in the absence of physical contact (e.g. disease transmission; Hamede *et al*. 2009), such as the diffusion of social information. We specifically strove to establish a calibration relationship – for the estimation of animal‐to‐animal distances from field‐recorded signal‐strength values – that would, as much as possible, account for the technological and biological complexities of an actual field deployment. Thus, rather than conducting ‘open‐field’ tests, with radio transmission between tags measured under ‘ideal’ conditions (e.g. open habitat and perfect antenna alignment), we developed procedures that enabled us to assess explicitly some of the inconvenient ‘noise’ that is caused by study subjects ranging across a diversity of habitat types (*cf*. Ceriotti *et al*. 2010; Marfievici *et al*. 2013).

Following a brief introduction of our study system, tool‐using New Caledonian crows *Corvus moneduloides*, we present in the following sections details of a multi‐step calibration exercise, comprising of: Step 1 – *field experiments* that measure how signal strength is affected by inter‐tag distance, as well as by a set of nuisance parameters (habitat type, tag height above ground and relative antenna orientation); Step 2 – a *theoretical model* that describes how radio waves are expected to propagate and attenuate under our set of experimental conditions; Step 3 – a *statistical model* that builds on our theoretical analyses, to estimate key parameters of our calibration relationship from our experimental data; and Step 4 – *computer simulations* that attempt a comprehensive system characterisation, by integrating our calibration results, basic assumptions about our study species’ behaviour and observations of wild, free‐ranging subjects. Although the reported analyses refer to a specific study species and deployment context, our approach can be applied to a multitude of other systems, providing a convenient ‘how to’ guide for future studies.

## Study system

### Proximity‐logging technology

**Figure 1:**
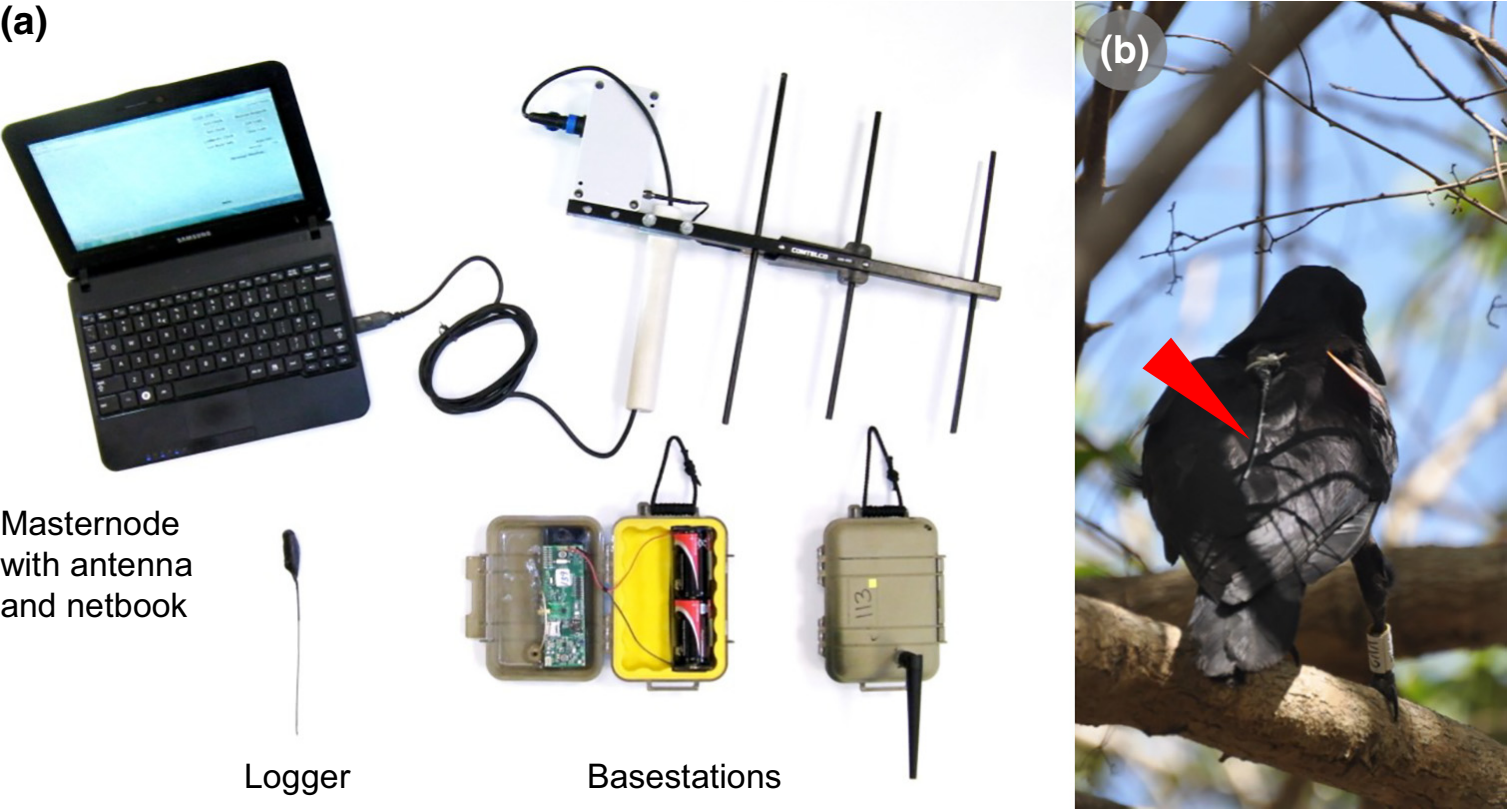
The study system. (a) Hardware components of an Encounternet proximity‐logging system: a logger; two basestations (one opened); and a masternode with Yagi antenna and netbook. For a basic description of system functionality, see main text. (b) Back view of a wild New Caledonian crow fitted with a harness‐mounted Encounternet proximity logger. Note how the antenna is projecting downwards from the back of the bird (red arrow), at approximately 45° against the horizontal (*cf*. Fig. 2b). Both panels are adapted from figures in Rutz *et al*. (2012).

Encounternet consists of three hardware components (‘nodes’ in WSN jargon; Fig. 1a). The core component is a set of animal‐mounted tags, which in our recent field deployment (Rutz *et al*. 2012; St Clair *et al*. in press) weighed 9**·**57 ± 0**·**05 g (mean ± SE; with a battery lifetime of several weeks), less than the 5% of subject body mass widely recommended for short‐term tagging studies (Bridge *et al*. 2011). Tags transmit ID‐coded radio pulses at a preprogrammed interval (in our field deployment, once every 20 s; see Rutz *et al*. 2012), while continuously receiving and logging pulses from nearby tags (range usually several tens of metres). Data stored in tag memory include (for a sample log, see Table 1): the ID codes of sending and receiving tags, the time of the received pulse and the ‘received signal strength indicator’ (RSSI) value of the received pulse, which is a measure of the power (in dB) of the received signal (for details, see Step 2). In order to use their limited on‐board memory (*ca*. 4000 logs) more efficiently, Encounternet tags can be programmed to average signal‐strength values across multiple consecutively received pulses, in which case a log includes data on the minimum, maximum and mean RSSI for the pulse sequence, as well as its beginning and end times (Table 1; see Step 4). In the following sections, we will first concentrate on estimating the relationship between RSSI and distance for single pulses, before discussing the consequences of pulse averaging. The second hardware component consists of larger receiver units (‘basestations’), which are placed at fixed locations within the study area, and can be programmed to both wirelessly receive and log radio pulses from nearby tags (range usually *ca*. 100 m) (Mennill *et al*. 2012), and to remotely download (and then clear) tags’ stored logs. These downloads are triggered when user‐specified conditions – such as a threshold number of logs held in tag memory – are met. Finally, hand‐held ‘masternodes’, which are operated with a directional Yagi antenna and a portable computer, allow wireless communication with tags and basestations, to remotely control their settings and retrieve any stored data.

Our work on tag‐to‐tag communication complements results from an earlier study (Galápagos sea lions *Zalophus wollebaeki*; Meise *et al*. 2013) and from another project that reported a detailed calibration for the detection of tags by fixed basestations (long‐tailed manakins *Chiroxiphia linearis*; Mennill *et al*. 2012; see also Snijders *et al*. 2014). Although calibrations were conducted for different species and habitats, taken together, these three studies provide a comprehensive ‘road map’ for how to prepare Encounternet systems for field deployment.

### Study species and study area

Our interest in the social structure of New Caledonian crows is centred on the question of how, and from whom, birds can potentially learn tool‐related information. For the operation of some social‐learning mechanisms (see Hoppitt & Laland 2013), it would be sufficient if ‘observers’ can see ‘demonstrators’ over relatively large distances, in the order of several tens of metres; under such circumstances, for example, crows may be attracted to profitable foraging patches or to particular plants from which tools can be made. In contrast, to observe details of tool‐manufacture and deployment behaviour directly, birds would need to be within a few body lengths of each other or several metres at most. Our calibration aimed at differentiating between these two scenarios, to enable investigations of how social dynamics might support the spread and maintenance of tool‐related (‘cultural’) information in wild crow societies (see Rutz *et al*. 2012; St Clair *et al*. in press).

We calibrated our Encounternet system for deployment in one of our long‐term study sites – a lowland section of *Melaleuca* spp. dry forest (Taro and Tabou valleys, Gouaro‐Déva; 21°33′ S, 165°19′ E). The local crow population has been subject of investigation since 2005 (see Bluff *et al*. 2010; Rutz *et al*. 2010) and consists of several resident breeding pairs with young and varying numbers of non‐breeding ‘floaters’ and short‐term visitors; overall, the basic social organisation appears comparable to that described for another study population (Holzhaider *et al*. 2011).

## Step 1 – Field experiments

The first step of our calibration exercise was to conduct field experiments in our designated deployment site, to measure tags’ radio transmission and reception under controlled, yet naturalistic, conditions. New Caledonian crows – like most other birds – move through complex 3D environments during their daily lives: they visit different types of habitat (of varying vegetation density; see above); use various vegetation strata (from ground level to canopy; Rutz *et al*. 2007); and engage in dynamic social interactions (so tags, and their antennae, will align in a multitude of different ways and may sometimes be shielded by their own bodies). Since all of these factors will affect the transmission of radio signals between crow‐mounted proximity loggers (and hence recorded RSSI values), as formally shown in Step 2 below, they were explicitly examined in our field experiments.

**Figure 2:**
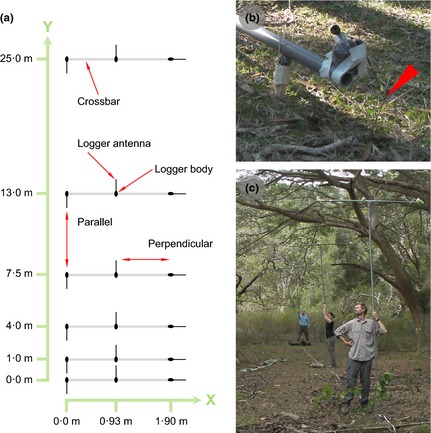
Field calibration experiments (Step 1). (a) Schematic top‐view of an ‘array’ of Encounternet proximity loggers, for assessing tag‐to‐tag radio communication in a variety of New Caledonian crow habitats. Three loggers are attached per 1·9‐m‐long PVC pole (‘crossbar’), with the orientation of loggers and the spacing of poles generating a large number of inter‐tag distances and relative antenna orientations (examples of parallel and perpendicular antennae shown; for further details, see main text). Poles at 1·0 m and 7·5 m distance (*y*) were only present in later trials. Note that dimensions are not to scale. (b) An Encounternet proximity logger attached to a ‘proxy’ crow (wrapped quail body), which is mounted as if ‘perched’ (red arrow shows antenna; *cf*. Fig. 1b). In this case, the array was deployed in the ‘ground’ condition in paperbark forest (0·1 m above ground, to mimic ground‐foraging crows). (c) An array deployed in the elevated (‘arboreal’) treatment condition in mixed gallery forest (4 m above ground, to mimic crows perched in the canopy).

We set up ‘arrays’ of 12 (and later 18) Encounternet tags that allowed us to assess simultaneously the radio communication of tags over 27 (59) different distances (ranging from 0·93 to 25·07 m) and relative antenna orientations (ranging from 0 to 180°) (see Fig. 2a). Tags were packaged in epoxy resin as if for field deployment, and taped to the back of proxy ‘crow’ bodies, consisting of shop‐bought, kitchen‐ready quails that had their body cavities stuffed with fresh chicken gizzards, before being sealed in rubber balloons to prevent dehydration. All tagged quails were mounted in an upright posture onto PVC poles, so that the tags’ antennae projected downwards at an angle of *ca*. 45° against the horizontal (Fig. 2b), as they would (on average) for harness‐mounted tags on perched or ground‐foraging New Caledonian crows (Fig. 1b). The position of individual tags within arrays was regularly changed between ‘trials’ (see below). Quails were discarded at the end of each test day at the latest, and Encounternet tags were replaced whenever masternode communication suggested a malfunction (see below).

We conducted a total of 24 trials, with each trial consisting of the deployment of an array in one of 5 different habitats in our study site (see Table 2), for 15 min elevated to *ca*. 4 m above the ground using hand‐held PVC poles (to mimic crows perching in the canopy; Fig. 2c), and (either before or after the ‘arboreal’ trial) placed for a further 15 min at *ca*. 0·1 m above ground on wooden support forks (to mimic ground‐foraging crows; Fig. 2b). Tags were programmed to transmit radio pulses once every 20 s and to store received data as single‐pulse log files. Hand‐held masternodes were used to send ‘start’ and ‘stop’ commands to all tags in the array and to download their data remotely after each trial.

**Table 2:** Habitat preferences of wild New Caledonian crows, as estimated from video footage recorded by crow‐mounted, miniature video cameras. Cell entries are estimates of the time (%) spent by 10 tagged crows in particular habitat and height combinations in the core study area (data from Rutz & Troscianko 2013). Crow position was scored in video footage as either ‘arboreal’ or on the ‘ground’, to correspond to the 4‐m and 0·1‐m categories used in the field experiments of Step 1

Habitat type	Arboreal (%)	Ground (%)
*Casuarina* spp.	3·0	0·4
fig trees (*Ficus* spp.)	13·4	1·7
mixed gallery forest (incl. *Aleurites moluccana*)	11·1	1·4
paperbark (*Melaleuca* spp.)	50·3	6·3
shrubs (incl. *Cordia dichotoma*)	11·1	1·4

After field work, we quality‐checked and cleaned the raw data as follows: we filtered out all logs (1·02%) that did not result from communication between tags, including those flagged up by system error messages; we retained only logs that were based on single pulses; and we discarded data that were recorded outside our 15‐min time window (so only data were used that had actually been recorded from a stationary array in a particular location, and not during array set‐up or transportation). The final data set comprised 91807 RSSI logs recorded across all trials (see data deposited in Dryad; Rutz *et al*. 2015).

## Step 2 – Theoretical model

To inform the statistical analysis of our empirical calibration data from Step 1, we develop a simple analytical model, from basic physical principles. The model takes into account properties of the tags and of the habitat between them.

### Propagation of radio waves

To begin with, we consider the propagation of radio waves from tag‐A to tag‐B, a distance *r* (in metres, m) apart. As the waves move through a particular habitat, treated as a homogeneous, isotropic medium, they lose intensity through two mechanisms: spherical spreading, leading to an inverse‐square dependence of received power *P*_*r*_ on distance *r*; and wave scattering and/or absorption, which produces an exponential decay in power. It is customary to relate the power *P*_*r*_ received by tag‐B to some (unspecified) reference power *P*_0_:

(eqn 1) $$\frac{P_{r}}{P_{0}}=C\frac{10^{\beta r/10}}{r^{2}}.$$

**Figure 3:**
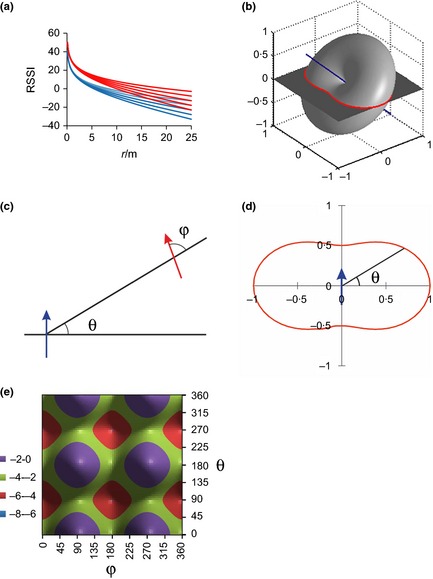
Features of the physical model (Step 2). (a) RSSI as a function of inter‐tag distance (see eqn 3 in the main text) for *K *=* *20 dB (blue) and *K *=* *30 dB (red). For each *K*, β takes values of −0·2 (top) to −1·0 (bottom) in decrements of 0·2. (b) Theoretical radiation pattern of a dipole antenna. The antenna axis (blue) is tilted at 45° to the horizontal, as it was for tags in our calibration experiment (Fig. 2b), and as it would be, on average, for tags mounted on wild crows (Fig. 1b). The radiation transmitted (and received) in a horizontal plane cutting through the centre of the antenna pattern at (0,0,0) is highlighted in red. (c) Angles used to describe the in‐plane radiation pattern between two communicating tags (arrows pointing in the ‘forward’ direction). (d) The in‐plane radiation pattern of a single tag, taken from the toric section highlighted in panel (b). (e) The in‐plane directivity function δ (in dB) as a function of the orientations (θ,φ) of two communicating tags (*cf*. panel c).

The absorption coefficient β (≤0) and the multiplicative factor *C* are functions of both the habitat *h* in which signals are being transmitted and the tags’ height above ground level (in our case: *z *=* *0·1 m; *z *=* *4 m). The RSSI recorded by tag‐B is the power ratio *P*_*r*_/*P*_0_ (expressed in decibels, dB):

(eqn 2) $$\text{RSSI}\equiv 10\log_{10}\left(\frac{P_{r}}{P_{0}}\right)=K+\beta r-20\log_{10}r$$

where *K *=* *10log_10_ *C*. Figure 3a illustrates that RSSI is a monotonically decreasing function of distance; more negative values of β, indicating a medium that absorbs or scatters more of the radiation, will produce more rapid decay of RSSI with increasing distance, while an increase in *K* will shift the graph vertically. Importantly, for any pair of parameters (*K*, β), the rate of change of RSSI is greatest at small inter‐tag distances *r*, a property that fundamentally affects researchers’ ability to estimate, for any proximity‐logging system, animal‐to‐animal distances from field‐recorded RSSI values. In addition to habitat and height, tag power and orientation will influence parameter *K*, as explored in detail in the following sections.

### Variation in tags’ power output

Any inherent variability in tag power – for example, due to subtle differences in transmitter components, antenna configurations or both – will affect the recorded RSSI (*cf*. Boyland *et al*. 2013). We used a large number of tags in our calibration experiment (see Step 1), but did not measure their power output directly. Data from another study using Encounternet technology (Mennill *et al*. 2012), however, suggest a range in between‐tag power differences of 1–2 dB, which should therefore be regarded as the best resolution we can achieve for *K* in our model.

### Relative orientation of communicating tag pairs

Each tag antenna acts both as a transmitter and as a receiver of radio waves (see Introduction), in an entirely reciprocal fashion. The antenna has an anisotropic radiation pattern, radiating (and receiving) more power to (and from) some directions than others. By design, a mounted tag acts as a dipole antenna, with a ‘doughnut’‐shaped power radiation pattern (Kenward 2001; Fig. 3b) – no radiation is produced (or received) along the axis of the antenna, and the direction of maximum power is perpendicular to this axis.

In the deployment of tags on wild birds, the relative orientation of the transmitter and receiver tags may confound an attempt to convert RSSI values into tag‐to‐tag distances; we return to this issue below. In our calibration experiment, the antenna orientations are known and can therefore be accounted for. To do this, we take the simplest model for the radiation pattern of a dipole antenna where the power radiated (received) at an angle α to the antenna axis is proportional to sin^2^α, with 0° ≤ α ≤ 180°. This model assumes that the antenna pattern is unaffected by the proximity of the bird on which it is mounted. In each calibration measurement, tag‐A and tag‐B are positioned at the same height *z*, mounted at 45° to the horizontal (see Step 1), so the only relevant part of the radiation pattern is the curve formed by the intersection of the horizontal plane and the tilted doughnut (Fig. 3b). Mathematically, this curve is described by $\cos\alpha =\frac{1}{\sqrt{2}}\sin\theta$, where θ is the angle shown in Figure 3c. Rearranging, we find that the in‐plane radiation pattern is *I*(θ) = ½(1 +  cos^2^ θ) (Fig. 3d). The maximum power, *I*(0) = 1, is radiated to the sides and is twice as much as the power radiated to the front and back; note that any absorption of radiation by the bird would diminish *I*(θ) in the ‘forward’ direction, centred on θ = 90°.

The receiving tag antenna will have an identical in‐plane radiation pattern, rotated an angle φ about the line‐of‐sight bearing from tag‐A to tag‐B (Fig. 3c). The effect on the received power *P*_*r*_ of the relative orientation of the two antennae is given by a simple product of their in‐plane radiation patterns, *D*(θ,φ) = ¼(1 + cos^2^θ)(1 + sin^2^φ), which has an additive effect on the RSSI signal (in dB) of δ(θ,φ) = 10 log_10_ *D*(θ,φ). The function δ(θ,φ) has a maximum of 0 dB (corresponding to *D *=* *1) when tags are side by side (so, for example, θ = 0°, φ = 90°), a minimum of −6 dB (*D *= ¼) when tags are aligned parallel (e.g. θ = 90°, φ = 0°) or antiparallel (e.g. θ = 90°, φ = 180°), and an average value of −2·5 dB, taken over all possible combinations of relative antenna orientations (Fig. 3e).

Given that we know the angles θ and φ for every calibration measurement, we can compute δ(θ,φ). We rewrite the parameter *K* in eqn 2 as *K *= κ + δ(θ,φ), where κ represents variation in the received power due only to height and habitat. Our physical model of the calibration experiment is then:

(eqn 3) $$\text{RSSI}=\kappa \left(h,z\right)+\beta \left(h,z\right)r-20\log_{10}r+\delta \left(\theta ,\varphi \right).$$

## Step 3 – Statistical model

### Model structure and fitting process

Equation 3 provides the basic structure for a statistical model that can be fitted to our empirical data from Step 1, to obtain estimates of, and levels of confidence in, our parameters κ and β for different habitats *h* and heights *z*. Since for each tag pulse (labelled with index i), the distance *r*_i_ between tags in the calibration array is known, we can construct our response variable as *y*_i_ = RSSI_i_ + 20log_10_ *r*_i_ without incurring problems of model endogeneity. Our final statistical model is:

(eqn 4) XXXXXXXXXXXXXXXXXXXXXXXXXXXX

where κ is the model intercept and β the effect of distance *r* on *y*. Although antenna angles (θ,φ)_i_ were known for our experimental data, the functional form of δ(θ,φ) contains assumptions (see Step 2) which we can test by including the fixed term γδ(θ,φ)_i_; if our assumptions were sound, we would expect γ = 1 in the fitted model.

The remaining terms are random effects that account for sources of variation over and above the effects of distance and relative tag orientation. The effect on RSSI of possible variation in the ability of individual tags to send and receive signals is modelled by *send*_i_ and *receive*_i_, respectively. The *exchange*(*h*_i_,*z*_i_)_i_ term is the effect of pairs of tags, with the variances in RSSI estimated separately for each habitat and height combination. *replicate*_i_ is the effect of replicate measurements of RSSI for tag pairs, that is, separately for the data where one tag was the transmitter and the other was the receiver and *vice versa*. Finally, ε_i_ are model residuals, or within‐replicate variance, accounting for all other effects. All random effects are assumed to be drawn from normal distributions with zero mean and estimated variances.

The model was implemented in the R package MCMCglmm (Hadfield 2010) and was run with default priors. We iterated the model for 130000 Gibbs sampling iterations, discarding the first 30000 iterations as a ‘burn‐in’ period. We subsequently retained every twentieth posterior sample, to obtain 5000 near‐independent samples of the joint posterior distribution of the model parameters (reported by summary.MCMCglmm()).

### Model results

**Figure 4:**
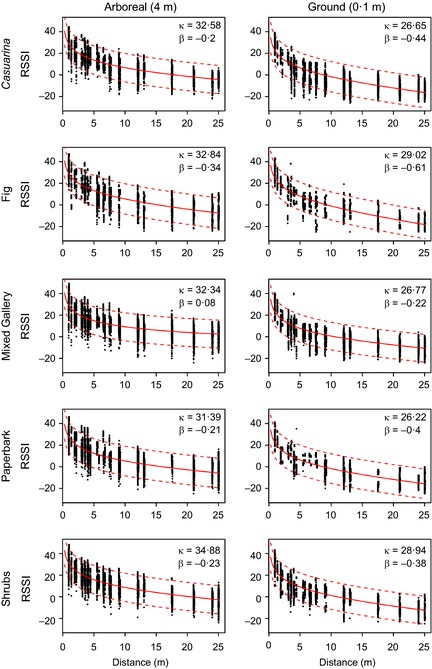
Fits of a mixed‐effects model to empirical calibration data (Step 3). The model produced estimates of key parameters κ (the model intercept) and β (the effect of distance on RSSI) of the calibration relationship (see eqns 3 and 4), separately for each habitat (rows) and height (columns) combination (*cf*. Table S1). Panels show the empirical (raw) data, with model fits indicated by red lines (lower bound, 2·5% quantile of the inferred total variance of the fitted model; upper bound, 97·5% quantile).

Figure 4 shows model fits for our experimental data from Step 1, illustrating the (combined) effects of habitat type (rows) and tag height above ground (columns) on RSSI. Overall, our physical model provided a good fit to the data, and as expected, γ was estimated to be close to 1 (1·099, with a 95% credible interval of 1·001–1·236), suggesting our simple dipole assumptions for the tag radiation pattern, excluding directional effects due to the presence of the bird (quail) body, were reasonable. Tags that were relatively good transmitters were also good receivers (correlation coefficient, 0·412, 0·062–0·637), which is likely a reflection of their antenna properties.

Model parameters κ and β varied considerably with habitat and height. Not surprisingly, relatively open habitats (mixed gallery forest) had higher β values (i.e. less pronounced signal attenuation with distance) than denser ones (fig trees or shrubs) (Fig. 4; Table S1), and for all habitats investigated, both κ and β values for the arboreal condition exceeded those estimated for the ground condition (compare Figs 3a and 4). It is clear from our statistical analyses that, for animals using a variety of habitats, there is no single function that relates RSSI reliably to inter‐animal distance, and that even within a particular habitat type, there is considerable variation in RSSI for any given distance. This unfavourable signal‐to‐noise ratio has implications for our ultimate goal of estimating inter‐bird distances from field‐recorded RSSI data (see Step 4).

## Step 4 – Computer simulations

In the final step of our calibration exercise, we need to pursue two objectives. First, since proximity loggers are (so far) unable to record contextual information for animal encounters, and thus measured RSSI values, it is necessary to establish a ‘master’ calibration relationship that ‘integrates’ information in a meaningful way across all relevant transmission scenarios (such as the 10 habitat–height combinations illustrated in Fig. 4). Second, we need to ‘invert’ this master calibration, in which distance (predictor) is related to RSSI (response), to enable conversion of field‐recorded RSSI (predictor) values into distance (response) estimates, or rather probability distributions of distances. While these two problems are difficult to tackle in a parametric statistical framework, it is reasonably straightforward to simulate the distribution of RSSI values one would expect to be generated by tags on a population of wild, free‐ranging crows, using: outputs from our statistical model (Step 3); additional information about our study system; and some basic assumptions.

### Parameterisation and assumptions

The basic rationale of our simulations is to place ‘crows’ (36 individuals) in a ‘study area’ (4000 × 4000 m) (numbers based on estimates for our population; see Rutz *et al*. 2010) and to note the RSSI values their ‘tags’ would record. For generating these RSSI values, we used the coefficients and variance estimates from our statistical model (Table S1), with the following additional assumptions about our study system. First, we assumed that the relative antenna orientation of tag pairs was unbiased, that is, in each simulated tag‐to‐tag pulse, we made each angle θ and φ equally likely. Second, we assumed that pairs of birds occupy the 10 habitat–height combinations with a frequency proportional to crows’ actual habitat preferences (Table 2), as estimated from footage obtained with crow‐borne video cameras in our study site in 2009–2010 (for details, see Troscianko 2012; Rutz & Troscianko 2013). This process of ‘weighting’ data according to independently observed crow behaviour minimises the impact of the variation of κ and β and is clearly preferable to an assumption that all habitats and heights are sampled uniformly.

**Figure 5:**
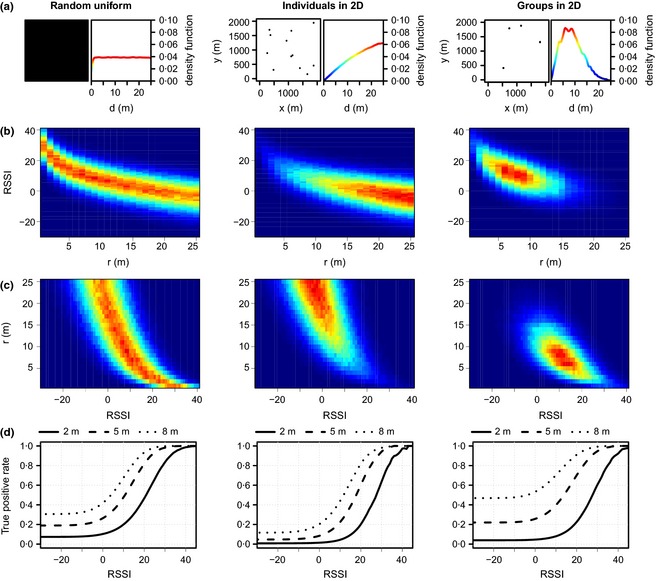
Computer simulations (Step 4), integrating information from calibration Steps 1–3. The three columns illustrate the effects of assuming different underlying distributions of inter‐bird distances: random uniform distribution (*left*); random individual locations in 2D space (*middle*); and distribution with a tendency of individuals to cluster in small groups (*right*). (a) An example of the simulated spatial distribution of birds and the resulting density distribution of inter‐bird distances. (b) Simulation of 1 million RSSI values, with the placement of points on the distance axis informed by the density function for each scenario. (c) The same data as in panel (b), with the axes inverted. (d) The proportion of points of a given RSSI, which occurred within a given distance threshold, plotted for a range of distance thresholds (solid line = 2 m; dashed line = 5 m; dotted line = 8 m). The chosen distance thresholds are for illustration purposes only, and estimates for particular RSSI cut‐offs – as reported in the main text – need to be derived from raw simulation outputs.

Finally, we have to make assumptions about the spatial distribution of crows in the study area, which determines tag‐to‐tag distances, and thus RSSI values. This may seem like a circular problem – after all, the point of deploying proximity loggers is to learn something about spatial distributions – but to remain indifferent to this issue is effectively to assume a uniform random distribution, in which any given inter‐animal distance is as likely to occur as any other. While it is useful to explore this default scenario (see left‐hand panels in Fig. 5), this is of course an implausible situation in nature; importantly for our purposes, it will tend to underestimate the proportion of large inter‐individual distances and may thus lead to overestimates of the frequency of relatively close associations. This limitation can be addressed by simulating random locations of individuals in two‐dimensional space (see middle panels in Fig. 5). For populations that are expected to have a clumped distribution – which includes the vast majority of social animals, and those which exploit patchy resources – this approach has the disadvantage that the proportion of relatively short inter‐individual distances will be underestimated, leading us to underestimate the occurrence of relatively close associations in the real world. Further refinements can be achieved by allocating simulated individuals to groups, where within‐group distances are on average smaller than between‐group distances (see right‐hand panels in Fig. 5). For our study system, this scenario best approximates biological reality, as it acknowledges the fact that New Caledonian crows move around in social groups, with the core social unit being the family (Rutz & St Clair 2012); as the group size for our simulations, we used 3 individuals as estimated by an independent radio‐tracking study (see Holzhaider *et al*. 2011), and the average ‘diameter’ of groups was set at 10 m, based on our own field observations. Having such prior knowledge of the study species’ spatial ecology is a distinct advantage; without this, it is advisable to explore how sensitive conversions are to varying prior estimates (i.e. distributions of inter‐individual distances), as illustrated in Figure 5.

### Interpreting real‐world data

Our simulations allow us to estimate the proportion of pulses at, or above, a given RSSI value that we would expect to occur over a given distance or less (see Fig. 5). In our simplistic ‘random uniform’ scenario, we estimate that 50% of pulses of an RSSI of 15 or greater will result from an inter‐tag distance of 3·51 m or less, while 95% of pulses will originate from within 14·51 m, with the corresponding values for our ‘individuals in 2D’ scenario being 6·51 m and 23·50 m, respectively. Finally, the ‘groups in 2D’ scenario, which represents the best characterisation of our study system we can achieve to date (see above), produces estimates of 4·74 m and 11·29 m, respectively. While the choice of the RSSI value used for *post hoc* filtering of field data sets is of course arbitrary, our analyses have shown that Encounternet enables reliable distance‐binning in our application; the value used here (RSSI ≥15) achieves our original goal of identifying ‘short‐range’ associations between crows, and distinguishing them from more distant encounters.

### Use of multi‐pulse averages

In systems where animals could potentially associate for protracted periods away from basestations, there is a danger that thousands of accumulated logs will eventually fill up the on‐board memory of tags; depending on settings, this will force them to either overwrite data or to cease logging, until once more within basestation reception range. To address this problem, Encounternet tags have an option to automatically average RSSI values across sequences of received pulses, generating RSSI_mean_ values (see Study system and Table 1). The maximum number of pulses which are averaged, and the time between pulses, are both programmable, allowing researchers to optimise the trade‐off between the danger of filling tag memory and the resolution of logged data.

Averaged values contain more information than single pulses, so they should in principle lead to better distance estimates; in fact, the large variance component for the *exchange* random effect in our statistical model of Step 3 suggests that there is considerable scope for such an ‘averaging’ effect (Table S1). In practice, RSSI_mean_ values should be interpreted slightly differently to single‐pulse RSSI values. First, because animals tend to move around relative to one another during encounters, their distance of closest approach will generally be much closer than the RSSI_mean_ indicates. This can be tested by investigating RSSI_mean_ and RSSI_max_ simultaneously (see St Clair *et al*. in press). Second, short encounters (those with a duration less than the programmed pulse interval multiplied by the number of pulses to be averaged) will tend to have low RSSI_mean_ values, because the pulses received as the birds come together at the beginning of the encounter, and move apart at its end, will tend to drag the mean downwards. This makes the use of RSSI_mean_ inherently conservative. Consequently, applying a filter of RSSI_mean_ ≥ 15 to field data (see St Clair *et al*. in press) will identify crow associations that were substantially closer than suggested by our single‐pulse estimates reported above.

## Conclusions

We have presented a calibration for a long‐range proximity‐logging system that takes into account the movement of tagged animals across heterogeneous environments. Our analyses confirm that, in our crow study system, it is possible to assign field‐recorded signal‐strength values reliably to predefined distance classes, which is key for probing the role of different social‐learning mechanisms (Rutz *et al*. 2012; St Clair *et al*. in press). We hope that our multi‐step procedure provides a generalisable guide to those working on other species. We can think of a range of refinements that would further increase classification accuracy; for example, future work could: expand the range of contexts from which calibration data are acquired, which for birds might include associations between pairs of tags where one is on the ground and the other in the canopy (→Step 1); measure the tags’ radiation pattern in an anechoic chamber (→Step 2); quantify experimentally the ground‐plane and shielding effects of animal bodies, allowing inter‐individual variation in body mass to be accounted for (→Step 2; see Naef‐Daenzer *et al*. 2005); refine the description of subjects’ movement patterns and habitat use to improve simulation results (→Step 4); conduct sensitivity analyses to determine the likely importance of relative antenna orientation, spatial distributions and habitat use (→Step 4); explicitly measure the performance of every tag before field deployment, for tag‐level corrections at the data analysis stage (→Step 4; *cf*. Boyland *et al*. 2013); integrate additional sensors into proximity loggers (such as GPS; Cagnacci *et al*. 2010), to enable context‐specific data conversion (→Step 4); and provide direct validation by attempting to observe tagged subjects during some of their encounters (→Step 4; Meise *et al*. 2013; *cf*. Shamoun‐Baranes *et al*. 2012), which would also allow quantitative assessment of the influence of subjects’ movements on RSSI, particularly when multi‐pulse averaging is used.

Although proximity logging has clear utility in some systems, it is important to acknowledge its limitations. Our study demonstrates that it would be a dubious exercise indeed to attempt converting individual RSSI values into precise individual distance estimates. As a general rule of thumb, the reliability of distance estimates will decrease with increasing habitat variability (i.e. if subjects use a wide range of habitats that differ in their signal‐attenuation properties), and distance ranges (i.e. if data need to be interpreted in the shallow, ‘fat’ tail of the calibration relationship; *cf*. Figs 4 and 5). These considerations are important, as they can inform decisions about whether proximity logging is the best tracking technology for a particular project (as opposed to, for example, GPS or PIT/RFID technology; see Krause *et al*. 2013), and if so, with which settings a given system should be deployed. The latter point refers to the distinction we made in the Introduction between short‐range systems with binary data recording and long‐range systems with *post hoc* filtering (as used here); the former may be somewhat easier to set up and operate, but only the latter are suitable for applications where researchers wish to map associations over a wider range of encounter distances. As in any biologging project, hardware and software settings should be chosen to optimise data quality and quantity given the constraints of battery life, memory size and tag mass. This optimisation is highly species and question specific: for example, when brief encounters are of interest, pulse rate may be maximised at the expense of battery life; when the species’ ecology hampers data retrieval, sampling rates and/or data compression may be adjusted at the expense of resolution; and when subjects’ behaviour is likely to be influenced by tagging, device mass can be reduced at the expense of both. In whatever form proximity logging is used, it is clear from our study, and earlier work (for Encounternet: Mennill *et al*. 2012; Meise *et al*. 2013; other WSNs: Ceriotti *et al*. 2010; Marfievici *et al*. 2013), that systems need to be calibrated specifically for each planned deployment.

While proximity‐logging systems require considerable resources for calibration, field deployment and operation, they enable fully automated, near‐real‐time collection of association data for entire animal populations, at unprecedented spatio‐temporal resolutions. These advances bring researchers tantalisingly close to mapping the ‘real’ social networks under investigation – one of the premises of the emerging field of ‘reality mining’ (Krause *et al*. 2013). At least in terrestrial applications, proximity logging is quickly becoming the method of choice for studying fine‐scale intra‐ and inter‐specific association patterns, in systems where direct observation or GPS tracking are not feasible.

## Supporting information


**Table S1.** Coefficients (posterior modes) of (a) fixed and (b) random effects, with 95% credible intervals, of the statistical model of Step 3, which was fitted to the empirical calibration data from Step 1.
