# Supplementary material for: Calibrating animal‐borne proximity loggers
Source: Methods Ecol Evol. 2015 May 6;6(6):656–67. doi: 10.1111/2041-210X.12370 (PMC4974916; doi:10.1111/2041-210X.12370)
Supplement: Supplementary file 1 — Table S1. Coefficients (posterior modes) of (a) fixed and (b) random effects, with 95% credible intervals, of the statistical model of Step 3, which was fitted to the empirical calibration data from Step 1. [file MEE3-6-656-s001.pdf]

Table S1. Coefficients (posterior modes) of (**a**) fixed and (**b**) random effects, with 95% credible intervals, of the statistical model of Step 3, which was fitted to the empirical calibration data from Step 1. Compare with Figure 4.

**model term**	**coefficient**	**credible interval**
(**a**) fixed effects		
κ, *Casuarina*, 4 m	32.584	31.679 – 34.067
κ, fig, 4 m	32.843	31.675 – 34.281
κ, mixed gallery, 4 m	32.336	31.387 – 33.449
κ, paperbark, 4 m	31.391	30.014 – 32.537
κ, shrubs, 4 m	34.877	33.708 – 35.996
κ, *Casuarina*, 0.1 m	26.646	25.349 – 27.956
κ, fig, 0.1 m	29.017	27.496 – 30.051
κ, mixed gallery, 0.1 m	26.766	25.64 – 27.921
κ, paperbark, 0.1 m	26.221	24.963 – 27.791
κ, shrubs, 0.1 m	28.943	27.583 – 30.032
β, *Casuarina*, 4 m	-0.203	-0.281 – -0.112
β, fig, 4 m	-0.341	-0.44 – -0.252
β, mixed gallery, 4 m	0.083	0.01 – 0.144
β, paperbark, 4 m	-0.205	-0.271 – -0.1
β, shrubs, 4 m	-0.23	-0.309 – -0.144
β, *Casuarina*, 0.1 m	-0.438	-0.536 – -0.349
β, fig, 0.1 m	-0.606	-0.693 – -0.507
β, mixed gallery, 0.1 m	-0.221	-0.3 – -0.144
β, paperbark, 0.1 m	-0.403	-0.5 – -0.299
β, shrubs, 0.1 m	-0.384	-0.45 – -0.28
γ	1.099	1.001 – 1.236
(**b**) random effects		
send	0.341	0.115 – 0.902
receive	0.285	0.071 – 0.759
exchange, *Casuarina*, 4 m	30.881	25.757 – 36.097
exchange, fig, 4 m	38.522	32.414 – 45.207
exchange, mixed gallery, 4 m	23.404	20.231 – 27.846
exchange, paperbark, 4 m	28.646	23.498 – 34.252
exchange, shrubs, 4 m	28.43	23.376 – 33.122
exchange, *Casuarina*, 0.1 m	39.895	33.258 – 46.456
exchange, fig, 0.1 m	35.237	29.047 – 41.519
exchange, mixed gallery, 0.1 m	32.783	27.513 – 37.846
exchange, paperbark, 0.1 m	40.639	33.566 – 47.861
exchange, shrubs, 0.1 m	33	28.038 – 39.424
replicate	1.765	1.653 – 1.873
ε, *Casuarina*, 4 m	5.938	5.77 – 6.13
ε, fig, 4 m	4.795	4.683 – 4.968
ε, mixed gallery, 4 m	11.043	10.806 – 11.365
ε, paperbark, 4 m	12.206	11.745 – 12.534
ε, shrubs, 4 m	7.674	7.443 – 7.89
ε, *Casuarina*, 0.1 m	4.27	4.134 – 4.391
ε, fig, 0.1 m	0.368	0.357 – 0.383
ε, mixed gallery, 0.1 m	2.52	2.454 – 2.598
ε, paperbark, 0.1 m	1.036	1.006 – 1.074
ε, shrubs, 0.1 m	1.831	1.77 – 1.877
